# Is the tide turning again for cephalosporin resistance in *Neisseria gonorrhoeae* in Europe? Results from the 2013 European surveillance

**DOI:** 10.1186/s12879-015-1013-x

**Published:** 2015-08-11

**Authors:** Michelle J. Cole, Gianfranco Spiteri, Susanne Jacobsson, Rachel Pitt, Vlad Grigorjev, Magnus Unemo, The Euro-GASP Network

**Affiliations:** Sexually Transmitted Bacteria Reference Unit, Microbiological Services, Public Health England, London, England United Kingdom; European Centre for Disease Prevention and Control, Stockholm, Sweden; WHO Collaborating Centre for Gonorrhoea and other STIs, National Reference Laboratory for Pathogenic Neisseria, Department of Laboratory Medicine, Microbiology, Faculty of Medicine and Health, Örebro University, Örebro, Sweden

**Keywords:** *Neisseria gonorrhoeae*, Gonorrhoea, Treatment, Antimicrobial resistance, Cefixime, Ceftriaxone, Surveillance, European Gonococcal Antimicrobial Surveillance Programme (Euro-GASP), Europe

## Abstract

**Background:**

The emerging resistance to the extended-spectrum cephalosporins (ESCs) in *Neisseria gonorrhoeae* together with increasing incidence of gonorrhoea cases in many countries have been global public health concerns. However, in recent years the levels of ESC resistance have decreased in several regions worldwide. We describe the European Gonococcal Antimicrobial Surveillance Programme (Euro-GASP) data from 2013, and compare them to corresponding data from 2009–2012.

**Methods:**

During 2013, *N. gonorrhoeae* isolates from 21 participating countries were examined. Antimicrobial susceptibility testing (Etest or agar dilution) was performed for cefixime, ceftriaxone, ciprofloxacin, azithromycin, spectinomycin and gentamicin. Statistical analyses were performed to identify significant changes in resistance between years and to investigate associations between patients with resistant gonococcal isolates and collected epidemiological variables.

**Results:**

In total, 93 (4.7 %) of 1994 isolates displayed resistance to cefixime, representing an increase compared to the 3.9 % detected in 2012 (p = 0.23). Cefixime resistance was detected in 13 (61.9 %) of the 21 countries. Cefixime resistance among men who have sex with men was only 1.2 %, compared to 5.6 % and 6.1 % in females and male heterosexuals, respectively. The univariate analysis confirmed that isolates resistant to cefixime were more likely to be from females (OR 4.87, p < 0.01) or male heterosexuals (OR 5.32, p < 0.01). Seven (0.4 %) isolates displayed ceftriaxone resistance (in addition to cefixime resistance) compared to three and 10 isolates in 2012 and 2011, respectively. All 93 isolates with cefixime resistance were additionally resistant to ciprofloxacin and 16 (17.2 %) were also resistant to azithromycin. Among all tested isolates (n = 1994), the ciprofloxacin resistance level (52.9 %) was higher than in 2012 (50.1 %; p = 0.08), and azithromycin resistance (5.4 %) increased since 2012 (4.5 %; p = 0.16).

**Conclusions:**

In 2013, the ESC resistance was again slightly increasing in Europe. This emphasises the importance of implementing the actions outlined in the European and additional response plans, particularly activities strengthening the surveillance of antimicrobial resistance. Ceftriaxone combined with azithromycin remains a satisfactory option for the first-line treatment of gonorrhoea. However novel antimicrobials (new derivatives of previously developed antimicrobials or newly developed antimicrobials) for effective monotherapy or at least inclusion in new dual antimicrobial therapy regimens (combined with previously developed antimicrobials or novel antimicrobials) will likely be required.

## Background

The *in vitro* resistance emerging in *Neisseria gonorrhoeae* internationally to the last remaining options for empiric first-line treatment of gonorrhoea, the extended-spectrum cephalosporins (ESCs) cefixime and ceftriaxone, which has translated into clinical treatment failures, has been well documented in recent years [1–14]. Some well-characterised multidrug-resistant *N. gonorrhoeae* (MDR-NG) strains have accounted for most of this *in vitro* and clinical resistance to the ESCs worldwide, such as the *N. gonorrhoeae* multi-antigen sequence type (NG-MAST) ST1407 or genetically closely related STs [3–8, 10, 12]. This developing situation along with the rising incidence of reported gonorrhoea cases in many particularly high-income countries [15–17], the associated morbidity of untreated gonorrhoea such as ectopic pregnancy and infertility, and the fact that gonorrhoea can substantially increase HIV transmission [18–20], make gonorrhoea a global public health problem.

*N. gonorrhoeae* has a well-documented history of acquiring and developing antimicrobial resistance (AMR) to all drugs used therapeutically for gonorrhoea [2] and it could therefore be predicted that *N. gonorrhoeae* would develop resistance to the ESCs. In this emergent situation, there have been significantly increased efforts aiming to retain gonorrhoea as a treatable infection. National and international action plans, such as the World Health Organization (WHO) global action plan [21, 22], the Centers for Disease Control and Prevention (CDC) response plan [23] and the European Centre for Disease Prevention and Control (ECDC) response plan to the threat of MDR-NG [24] have emphasised the need to scale up AMR surveillance globally, the need for updating national and international gonorrhoea management guidelines to include introduction of dual antimicrobial therapy [25], and information dissemination about the situation to healthcare professionals, patients and the public. AMR in *N. gonorrhoeae* has also been frequently highlighted as a major concern in the broader AMR reports and strategies [26–28].

In the European Union (EU)/European Economic Area (EEA), surveillance of AMR in *N. gonorrhoeae* is performed through the European Gonococcal Antimicrobial Surveillance Programme (Euro-GASP) [24, 29, 30]. The programme documented a statistically significant increase in cefixime resistance from 2009 (5.1 %) to 2010 (8.7 %), however after 2010 the cefixime resistance levels started to decrease to a low of 3.9 % in 2012 [29, 30]. Similar decreases in the resistance to ESCs have also been documented in other regions [15, 16, 31–36].

The aims of the present study were to describe the Euro-GASP data from 2013, and compare them to the Euro-GASP data from 2009–2012.

## Methods

### *Neisseria gonorrhoeae* isolates and the Euro-GASP

During 2013, *N. gonorrhoeae* isolates from 21 participating countries were examined in the Euro-GASP (Table 1). Number of countries participating in Euro-GASP increased over time: in 2009, 16 countries participated in the Euro-GASP; Cyprus, Hungary, Ireland and Norway joined in 2010; Iceland joined in 2013; Romania participated only in 2010 and 2011. Isolates were collected during two time periods; from April to May, and from October to November. Nine (43 %) of the countries participating in 2013 followed a centralised testing model, i.e., all antimicrobial susceptibility testing was performed centrally (in laboratories at Public Health England or Örebro University Hospital, Sweden) by the Etest for cefixime and ceftriaxone and agar dilution breakpoint method for ciprofloxacin, azithromycin and spectinomycin, and the full agar dilution method for gentamicin. The remaining 12 (57 %) countries followed a decentralised testing model, that is, the antimicrobial susceptibility testing using the Etest or agar dilution method was performed in their own national reference or local laboratory (Table 1). To ensure quality and comparability of data, all countries performing decentralised testing fulfilled strict quality criteria, all countries participated in an annual external quality assessment programme, and all countries used identical international reference strains for quality controls. Further details regarding the Euro-GASP have been published elsewhere [29]. The resistance breakpoints stated by the European Committee on Antimicrobial Susceptibility Testing (EUCAST) were used (cefixime/ceftriaxone minimum inhibitory concentration (MIC) > 0.125 mg/L, azithromycin MIC > 0.5 mg/L and ciprofloxacin MIC > 0.06 mg/L) [37]. The following epidemiological variables were collected and subsequently categorised as follows: age (< 25 years or ≥ 25 years), sexual orientation and gender (men who have sex with men (MSM), male heterosexuals and all women), previous gonorrhoea (yes or no), and concurrent chlamydial infection or no chlamydial infection.

**Table 1:** Resistance to cefixime, ciprofloxacin and azithromycin in *Neisseria gonorrhoeae* isolates from 21 EU/EEA countries, 2013

Country	Antimicrobial									Method of testing
	Cefixime			Ciprofloxacin			Azithromycin			
	No. resistant	No. tested	%	No. resistant	No. tested	%	No. resistant	No. tested	%	
Austria	7	109	6.4	78	109	71.6	6	109	5.5	Centralised
Belgium	7	110	6.4	62	110	56.4	2	110	1.8	Decentralised – MIC
Cyprus	0	9	0	8	9	88.9	3	9	33.3	Centralised
Denmark	13	110	11.8	64	110	58.2	10	110	9.1	Decentralised – Etest
France	4	112	3.6	50	112	44.6	0	112	0	Decentralised – Etest
Germany	13	101	12.9	64	101	63.4	4	101	4	Centralised
Greece	11	75	14.7	54	75	72	15	66	22.7	Decentralised – Etest
Hungary	6	88	6.8	60	88	68.2	2	88	2.3	Centralised
Iceland	0	5	0	2	5	40	0	5	0	Decentralised – Etest
Ireland	0	103	0	27	103	26.2	3	103	2.9	Decentralised – Etest
Italy	0	100	0	63	100	63	1	100	1	Decentralised – Etest
Latvia	1	38	2.6	10	38	26.3	6	38	15.8	Centralised
Malta	0	31	0	11	31	35.5	0	31	0	Decentralised – Etest
The Netherlands	0	139	0	48	139	34.5	2	139	1.4	Decentralised – Etest
Norway	5	112	4.5	89	112	79.5	12	112	10.7	Centralised
Portugal	0	110	0	52	110	47.3	20	110	18.2	Decentralised – Etest
Slovenia	1	73	1.4	46	73	63	0	73	0	Centralised
Slovakia	5	110	4.5	52	110	47.3	2	110	1.8	Centralised
Spain	18	119	15.1	78	119	65.5	10	119	8.4	Decentralised – MIC
Sweden	0	100	0	60	100	60	9	100	9	Decentralised – Etest
United Kingdom	2	240	0.8	77	240	32.1	1	240	0.4	Decentralised – MIC
**Total**	93	1994	4.7	1055	1994	52.9	108	1985	5.4	
**95** **%** **CI**			3.8 – 5.7			50.7 – 55.1			4.5 – 6.5	

EU/EEA, European Union/European Economic Area; MIC, minimum inhibitory concentration using Etest and/or agar dilution method; CI, confidence interval of the mean %

### Statistical analysis

The statistical significance of any changes in the proportion of isolates with resistance to tested antimicrobials between years was determined by the Z-test or Fisher’s exact test if cell numbers were less than 5. A univariate analysis and multivariable logistic regression analyses of odds ratios (OR) with 95 % confidence intervals (CI) were calculated to investigate associations between patients infected with an isolate displaying resistance to cefixime, ciprofloxacin or azithromycin, and the collected epidemiological variables. A Pearson χ^2^-test was used to test if these odds ratios were significantly different from one, with a P-value of < 0.05 indicating significance. Statistical analysis was performed in STATA v12.1 (StataCorp LP, TX, USA).

### Ethics

The present study was a surveillance study, using data from the Euro-GASP which is implemented through the ECDC Framework Contract No. ECDC/2013/015. All examined gonococcal isolates were cultured and preserved as part of the routine diagnostics (standard care) and no patient identification information was available in the present study. Ethical approval was therefore not required.

## Results

**Fig. 1:**
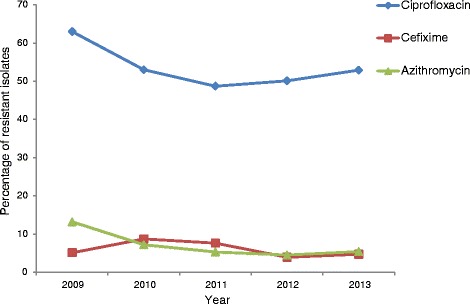
Azithromycin, cefixime and ciprofloxacin gonococcal resistance trends in the EU/EEA, 2009–2013

The results of all the antimicrobial susceptibility testing are summarised in Table 1. Of the 1994 examined *N. gonorrhoeae* isolates collected in 21 EU/EEA countries in 2013, 4.7 % (93 isolates) displayed resistance to cefixime (Table 1, Fig. 1). This represented a slight, but not statistically significant, increase compared to the 3.9 % of resistant isolates detected in 2012 (Z-test, p = 0.233). Cefixime resistance was detected in 13 (61.9 %) of the 21 countries, and in these countries cefixime resistance levels ranged from 0.8 % (United Kingdom) to 15.1 % (Spain) (Table 1). Nineteen (1.0 %) isolates had a MIC of cefixime of ≥ 0.5 mg/L compared to three isolates in 2012, and 13 isolates in 2011 (Fisher’s Exact test p = 0.001 and p = 0.379, respectively). These 19 isolates were from Spain (n = 7), Denmark (n = 3), Germany (n = 3), Austria (n = 2), Greece (n = 1), Hungary (n = 1), Slovakia (n = 1), and United Kingdom (n = 1). Seven (0.4 %) isolates displayed ceftriaxone resistance (six from Spain and one from Germany; all were additionally resistant to cefixime) compared to three isolates and 10 isolates in 2012 and 2011, respectively. All 93 isolates with cefixime resistance were additionally resistant to ciprofloxacin and 16 (17.2 %) were also resistant to azithromycin. Finally, of the seven ceftriaxone-resistant isolates, one (0.05 %) was also resistant to azithromycin and five (0.3 %) had a MIC (0.5 mg/L) exactly at the breakpoint for resistance.

**Fig. 2:**
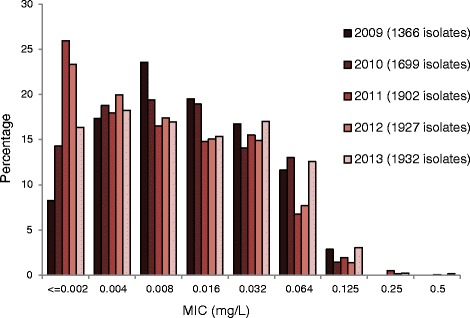
Ceftriaxone MIC distributions for *Neisseria gonorrhoeae* isolates in EU/EEA during 2009–2013

Furthermore, the MIC distribution for ceftriaxone in 2013 compared to the ones in 2011 and 2012 showed a decreased proportion of highly susceptible gonococcal isolates (MIC ≤ 0.002 mg/L) as well as increased proportions of isolates with higher MICs such as 0.064 mg/L and 0.125 mg/L (Fig. 2), which is exactly at the resistance breakpoint.

Overall, the ciprofloxacin resistance level of 52.9 % detected in 2013 was slightly higher than the 50.1 % of resistance detected in 2012 (Z-test, p = 0.082), continuing the gradually increasing trend observed since 2011 (Fig. 1). For the first time since 2008, azithromycin resistance increased, although the increase was not statistically significant (4.5 % in 2012, 5.4 % in 2013; Z-test, p = 0.159). However, only one isolate displayed high-level resistance to azithromycin (MIC ≥ 256 mg/L; collected in Ireland); high-level azithromycin-resistant isolates were also detected in 2006 (n = 1), 2007 (n = 4), 2011 (n = 2) and 2012 (n = 3). Finally, the modal MIC of gentamicin was 8 mg/L (MIC range: from 1 to 16 mg/L) and no resistance to spectinomycin (MIC range: from 0.5 to 64 mg/L) was demonstrated.

In Table 2, the number of patients with isolate susceptibility data (number of resistant isolates) linked with epidemiological data is summarised. In the univariate analysis, the only significant associations with cefixime resistance were being male heterosexual (OR male heterosexual vs. MSM 5.32, CI 2.12–13.3, p = 0.0001) or female (OR female vs. MSM 4.87, CI 1.89–12.6, p = 0.0003). For azithromycin, the univariate analysis revealed associations between azithromycin resistance and male heterosexuals (OR male heterosexual vs. MSM 2.39, CI 1.21–4.69, p = 0.0094). Associations were also observed between ciprofloxacin resistance and higher age (OR ≥ 25 years vs. < 25 years 1.36, CI 1.12-1.66, p = 0.0022), male heterosexuals (OR male heterosexual vs. MSM 1.7, CI 1.3–2.24, p = 0.0001), females (OR females vs. MSM 1.34, CI 1.01–1.79, p = 0.0437) and no concurrent chlamydia infection (OR no concurrent chlamydia vs. concurrent chlamydia 1.44, CI 1.03-2.02, p = 0.0313). In the ciprofloxacin multivariable analysis, the only significant associations remaining were for ciprofloxacin resistance and being male heterosexual (aOR: 1.57, CI 1.13–2.18, p = 0.007), and ciprofloxacin resistance and no concurrent *C. trachomatis* infection (aOR: 1.57, CI 1.09-2.24, p = 0.016).

**Table 2:** Number of patients with isolate susceptibility data (number of resistant isolates) linked with epidemiological data, i.e., sexual orientation, age group and concurrent chlamydial infection

	Total number of patient variables (no. resistant)						
	MSM	Male heterosexuals	Females	≥25 years	<25 years	Concurrent chlamydia	No concurrent chlamydia
Azithromycin	494 (14)	369 (24)	302 (16)	1391 (72)	553 (35)	183 (3)	658 (20)
Ciprofloxacin	496 (210)	376 (209)	302 (150)	1399 (769)	554 (262)	183 (69)	658 (307)
Cefixime	496 (6)	376 (23)	302 (17)	1399 (68)	554 (22)	183 (3)	658 (13)

MSM, men who have sex with men

## Discussion

The 2013 Euro-GASP data show that the encouraging recent trends of decreasing cefixime, ceftriaxone, ciprofloxacin and azithromycin resistance in *N. gonorrhoeae* across the EU/EEA region have not been maintained, although the increases observed in 2013 were not statistically significant. Further data are needed to establish if the increasing trend will continue. The 2013 data emphasize the need for continued expansion and improvement in quality-assured surveillance of gonococcal AMR and treatment failures, as described in the European and other response plans [21–24]. The data also stress that widespread implementation of current European management guidelines [25] or similar therapeutic regimens [38, 39] recommending ceftriaxone in combination with azithromycin for first-line treatment of all cases of uncomplicated gonorrhoea, remains crucial.

As well as decreases in the level of resistance to ESCs in the EU/EEA from 2010 to 2012, decreases have also been observed in other regions with well-developed surveillance programmes globally [15, 16, 31, 33–36]. Furthermore, despite some rare ESC treatment failures verified in recent years [3–14], the anticipated number of reported gonorrhoea treatment failures has not yet materialised, which could also be partly due to a lack of identification and under-reporting of treatment failures. The decrease in ESC resistance in Europe (2010–2012) and in several additional regions is an extremely interesting and perplexing situation as decreasing resistance trends have never been documented for any previously used antimicrobial in *N. gonorrhoeae*. The situation is even more confusing as similar decreasing trends have been witnessed in countries that have not used cefixime as any main therapeutic agent or have not changed their recommended treatment regimens [31, 33, 35, 36]. The reasons explaining all these decreases in ESC resistance are most likely multi-factorial and complex. A number of recent changes in the diagnostics and management of gonorrhoea may have contributed to the decreases in ESC resistance. In many countries, for example, increased use of more sensitive molecular diagnostics and increased testing of extra-genital sites, including the pharynx, among MSM might have contributed to reducing the reservoir of strains such as the NG-MAST ST1407 as effective diagnostics allows the administration of appropriate antimicrobial therapy; implementation of updated management guidelines recommending dual antimicrobial therapy could similarly have more effectively targeted resistant strains; education to healthcare professionals is also likely to have contributed to a more effective implementation of testing and treatment guidelines. The decrease in ESC resistance may also be due to an epidemiologic replacement of the MDR-NG ST1407 clone by other STs, which might potentially have been affected also by some type of partial immunity to the widely spread ST1407 from prior infection (17 % - 21 % of patients reported having had a previous gonorrhoea infection during years 2009 – 2013 [29]). Clearly, sufficient understanding regarding the gonococcal population dynamics, including epidemiologic curves for single strains, is lacking. In the Euro-GASP, a molecular typing study examining the 2013 isolates and associated AMR and epidemiologic data is currently underway. This study should further elucidate the perplexing situation with the initially decreasing ESC resistance trends that started to once again increase in 2013.

It is also crucial to emphasize that the resistance level to both cefixime and ciprofloxacin is higher in the heterosexual community. In 2013, cefixime resistance was significantly associated with heterosexual orientations: among MSM only 1.2 % of isolates were resistant to cefixime, compared to 5.6 % and 6.1 % in females and male heterosexuals, respectively. In the Euro-GASP, this trend was also earlier identified analysing material from 2009 to 2011 [40]. Due to the severe complications and sequelae resulting from ascending infection, the risk of untreated or inappropriately treated gonorrhoea is of particular concern in women where the infection is also much harder to diagnose and is often asymptomatic in nature. Hopefully, the ongoing molecular typing study examining the 2013 European gonococcal isolates and associated AMR and epidemiologic data will be able to further elucidate also the associations between AMR and sexual orientation, as well as additional epidemiological variables.

The main inherent limitations in the Euro-GASP have been detailed previously in the European response plan [24], and mostly include issues regarding the number and representativeness of gonococcal isolates and associated patients. Efforts are underway to address these limitations: in 2014, several additional countries have joined the Euro-GASP, an increased number of isolates with associated epidemiologic metadata are available from many countries, and a comprehensive review of the longitudinal AMR and epidemiological surveillance data to identify areas where representativeness needs to be improved is in progress. Nevertheless, in 2013, 21 (68 %) of the 31 EU/EEA countries were already participating in the Euro-GASP, which should provide a relatively effective evidence base for the gonococcal AMR situation in the EU/EEA region.

## Conclusions

Even though slight decreases in the ESC resistance were documented in recent years in Europe and several other regions globally, in 2013 the ESC resistance was once again increasing in Europe. Overall, this might be a time of ‘calm before the storm’ and efforts to keep gonorrhoea as a treatable infection need to be sustained. *N. gonorrhoeae* has already shown its capacity to develop high-level resistance to ceftriaxone [2, 6, 9] and, based on the well-documented history regarding all antimicrobials previously used for gonorrhoea treatment, it is likely that it is only a matter of time before ceftriaxone-resistant strains with sufficient biological fitness emerge and start to spread internationally. National and international surveillance of *N. gonorrhoeae* AMR (including collection of appropriate associated epidemiologic metadata), treatment failures and ideally also antimicrobial use/misuse, as well as implementation of recommended dual antimicrobial treatment regimens (ceftriaxone plus azithromycin) needs to be maintained and in several areas further strengthened to provide more reliable evidence for policy makers and to inform antibiotic prescription protocols. The work outlined in the European [24] and additional response plans [21–23] needs to be further implemented, and a close collaboration between the Euro-GASP and additional GASPs internationally [41] in liaison with the WHO Global GASP is crucial. The number of cases of gonorrhoea reported to the ECDC has been increasing annually since 2008; 52,995 cases were reported in 2013, which is an 11 % increase from the previous year (47,641 cases in 2012) [42]. This increasing gonorrhoea incidence, along with the slight rise of ESC resistance described in this study, confirms the importance of antimicrobial susceptibility surveillance for this disease. Presently, ceftriaxone combined with azithromycin remains a satisfactory option for the first-line treatment of gonorrhoea. However, novel antimicrobials (new derivatives of previously developed antimicrobials or newly developed antimicrobials) for effective monotherapy or at least inclusion in new dual antimicrobial therapy regimens (combined with previously developed antimicrobials or other novel antimicrobials) will ultimately be required if the upturn seen in 2013 is sustained into future years.
